# Supplementary material for: Using linear measurements to diagnose the ecological habitat of Spinosaurus
Source: PeerJ. 2024 Jun 13;12:e17544. doi: 10.7717/peerj.17544 (PMC11180429; doi:10.7717/peerj.17544)
Supplement: Supplemental Information 3 [file peerj-12-17544-s003.docx]

**Smart and Sakamoto 2023 Supplemental Materials**

**Supplemental methods:**

**Software used:** ImageJ v. 1.53 (Abràmoff et al., 2004) and PAST v. 4.03 (Hammer et al., 2001)

**File formats:** Skull images were stored variously as .JPG and .PNG files, main data was stored as a .CSV sheet, within a .XLSX workbook, and analysis was conducted in a .DAT file.

For full references, see main text.

**Comments on Figure 1a:**

The most striking feature of the resulting morphospace plot is the relative positions of spinosaurids from other theropods, and their proximity to marine mosasaurs and plesiosaurs (the plesiosaur *Pliosaurus* *kevani* had PC scores most similar to *Spinosaurus*) (figure 1c). Wading birds (storks and herons) display highly conserved skull morphology, and a more specialised condition than *Spinosaurus*, which in turn shows a more specialised morphology than *Baryonyx* and *Suchomimus* when compared to other theropods.

The assignments of *Tyrannosaurus* (theropod) and *Plotosaurus* (mosasaur) to Cluster 2 are seemingly influenced by proportionally longer skulls and higher orbits than related taxa in other clusters. Both are highly derived members of their lineages. Kruskal-Wallis testing showed the median value of geometric means differed between the clusters (χ^2^ = 9.79, *p* < 0.01) Despite distinct median values, there is a strong overlap between the ranges of each cluster.

**Supplemenetal results:**

All tests were conducted in PAST v. 4.03 (Hammer et al., 2001) using the file ‘*Smart and Sakamoto PAST Analysis.dat’*

**Test:** One-way Permanova (Clade)

**Input**:

scaled_skull_length, scaled_skull_height, scaled_skull_width, scaled_naris_to_anterior_margin, scaled_naris_to_dorsal_margin, scaled_orbit_to_dosal_magin, clade (group)

**Settings**:

Similarity index: Euclidian, Permutation N: 9999, Pairwise: Bonferroni-corrected p values.

**Results**:

Overall F = 18.69, *p* (same) < 0.0001.


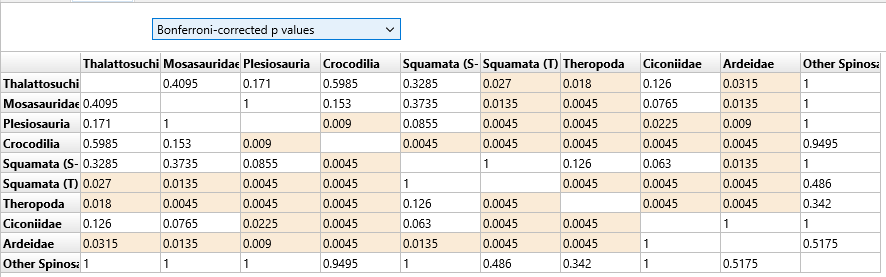


**Test:** One-way Permanova (Ecology)

**Input:**

scaled_skull_length, scaled_skull_height, scaled_skull_width, scaled_naris_to_anterior_margin, scaled_naris_to_dorsal_margin, scaled_orbit_to_dosal_magin, ecology (group) (all spinosaurids excluded).

**Settings:**

Similarity index: Euclidian, Permutation N: 9999, Pairwise: Bonferroni-corrected p values.


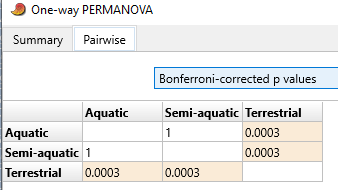
**Results:**

Overall F = 11.22, *p* (same) < 0.0001.

**Test:** Cluster analyses – K-means

**Input:**

scaled_skull_length, scaled_skull_height, scaled_skull_width, scaled_naris_to_anterior_margin, scaled_naris_to_dorsal_margin, scaled_orbit_to_dosal_magin

**Settings:**

Groups: 3

**Results:** See Figure 1 in main text.

**Test:** Cluster analyses - Classical

**Input:**

scaled_skull_length, scaled_skull_height, scaled_skull_width, scaled_naris_to_anterior_margin, scaled_naris_to_dorsal_margin, scaled_orbit_to_dosal_magin

**Settings:**

Algorithm: Ward’s Method, similarity index: Euclidean, two-way: no, constraints: none, boot N: 9999. Performed on all scaled and centred Mosimann logged values for the measurement variables.

**Results:**

Cophen correlation: 0.6675. See Figure 2 in main text.

**Test:** One-way ANOVA (k-means geometric means)

**Input:**

geometric_mean, K_means_cluster (group)

**
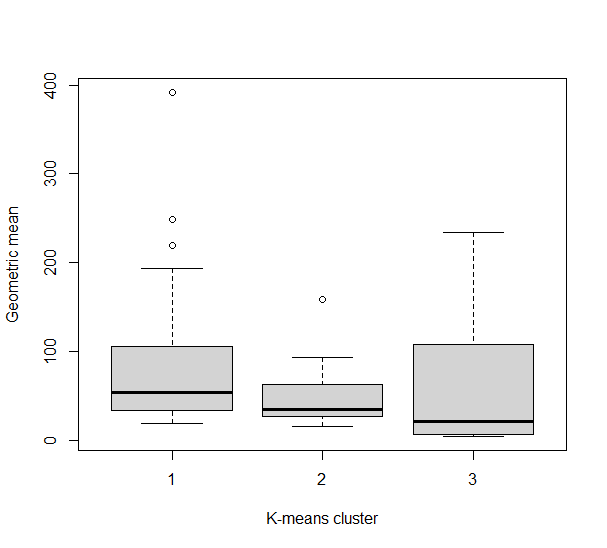
Settings:**

NA


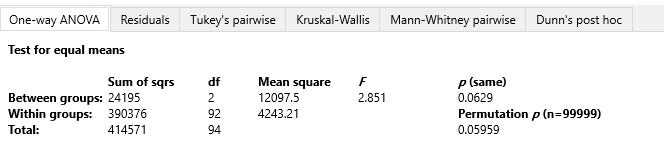
**Results:**

**Test:** PCA - Clades

**Input:**


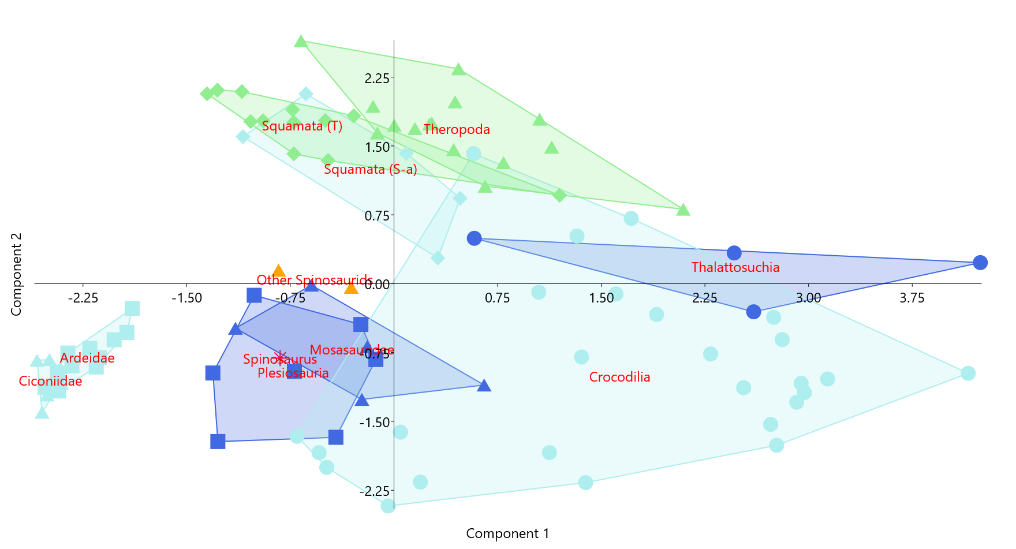
scaled_skull_length, scaled_skull_height, scaled_skull_width, scaled_naris_to_anterior_margin, scaled_naris_to_dorsal_margin, scaled_orbit_to_dosal_magin, clade (group)

**Settings:**

Matrix: Var-covar, Groups: Disregard, Missing Values: NA, Bootstrap N: 9999

**Results:**

See *‘Smart and Sakamoto PAST Analysis.dat’*


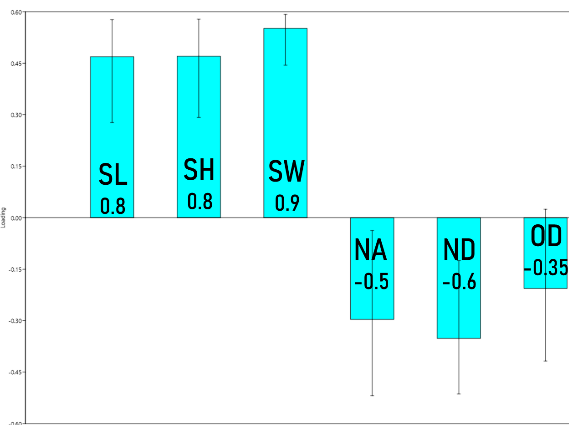
**
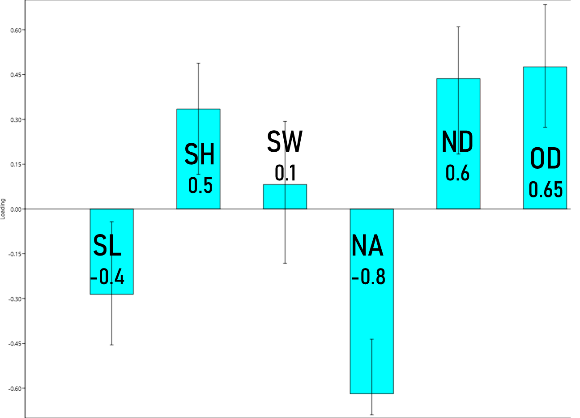
PC 1 (left) & 2(right) Loadings:** (Coefficients displayed by bar height, correlation displayed within bars)

| **PC 1-6 loadings coeffcients** | PC 1 | PC 2 | PC 3 | PC 4 | PC 5 | PC 6 |
| --- | --- | --- | --- | --- | --- | --- |
| scaled_skull_length | 0.46901 | -0.2859 | 0.21289 | 0.75262 | 0.16397 | 0.24421 |
| scaled_skull_height | 0.47037 | 0.33452 | -0.03705 | -0.3859 | 0.67006 | 0.25995 |
| scaled_skull_width | 0.55177 | 0.081821 | -0.21827 | -0.21041 | -0.69354 | 0.34052 |
| scaled_naris_to_anterior_margin | -0.29652 | -0.61856 | 0.028503 | -0.29261 | 0.094415 | 0.65887 |
| scaled_naris_to_dorsal_margin | -0.35189 | 0.43661 | -0.57597 | 0.39293 | 0.051403 | 0.44359 |
| scaled_orbit_to_dosal_magin | -0.20635 | 0.47583 | 0.75704 | 0.018767 | -0.17772 | 0.35491 |

| **PC 1-6 loadings correlations** | PC 1 | PC 2 | PC 3 | PC 4 | PC 5 | PC 6 |
| --- | --- | --- | --- | --- | --- | --- |
| scaled_skull_length | 0.79861 | -0.38526 | 0.19609 | 0.41331 | 0.067179 | 7.08E-11 |
| scaled_skull_height | 0.81485 | 0.45862 | -0.03472 | -0.21561 | 0.27929 | 7.66E-11 |
| scaled_skull_width | 0.92603 | 0.10867 | -0.19815 | -0.11389 | -0.28006 | 9.73E-11 |
| scaled_naris_to_anterior_margin | -0.51063 | -0.84298 | 0.026551 | -0.16251 | 0.03912 | 1.93E-10 |
| scaled_naris_to_dorsal_margin | -0.58935 | 0.5787 | -0.52182 | 0.21225 | 0.020715 | 1.26E-10 |
| scaled_orbit_to_dosal_magin | -0.34684 | 0.63295 | 0.68833 | 0.010174 | -0.07188 | 1.02E-10 |

**Test:** PCA - Ecology

**Input:**

scaled_skull_length, scaled_skull_height, scaled_skull_width, scaled_naris_to_anterior_margin, scaled_naris_to_dorsal_margin, scaled_orbit_to_dosal_magin, ecology (group) (classification for spinosaurids: “?”)

**Settings:**

Matrix: Var-covar, Groups: Disregard, Missing Values: NA, Bootstrap N: 9999

**Results:**


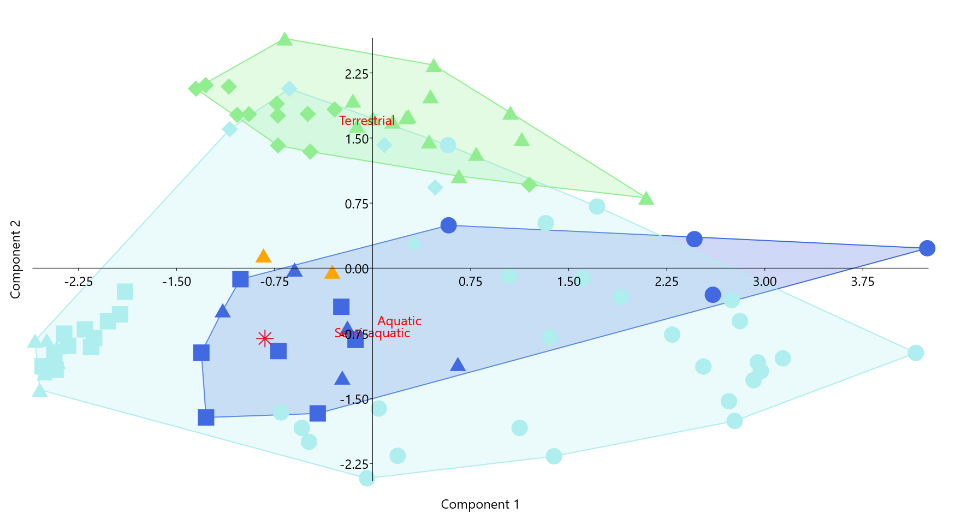
*
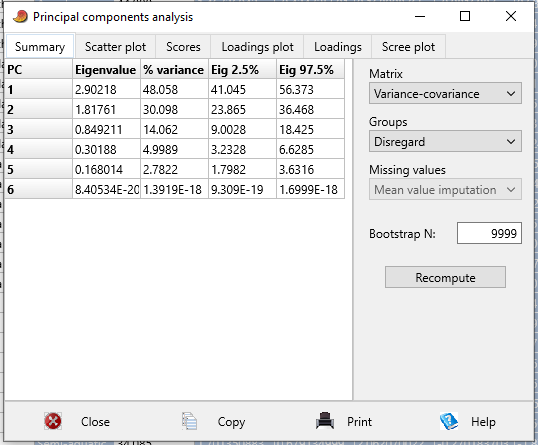
*See *‘Smart and Sakamoto PAST Analysis.dat’*

**Note:** PC loadings coeffcients and correlations are equal to the clade PCA, as this is unaffected by grouping variable.

**Test:** Spearman’s Corellation - (PC1 and Geometric mean)

**Inputs:**

PC1, geometric_mean

**Settings:**

NA

**Results:**

*ρ*_93_ = 0.0899, *p* = 0.386

**Test:** Kruskal-Wallis test - (k-means)

**Inputs:**


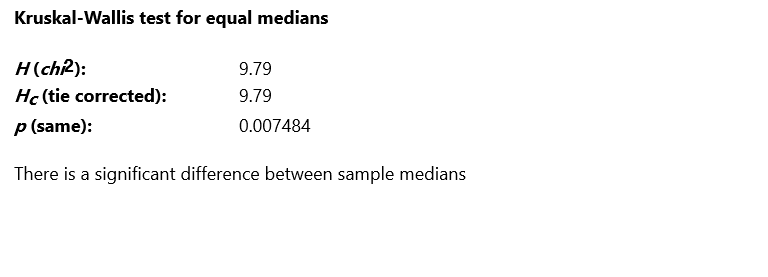
scaled_skull_length, scaled_skull_height, scaled_skull_width, scaled_naris_to_anterior_margin, scaled_naris_to_dorsal_margin, scaled_orbit_to_dosal_magin, K_means_cluster (group)

**Settings:**

NA

**Results:**

**Test:** Shapiro-Wilk test - (k-means)

**Inputs:**


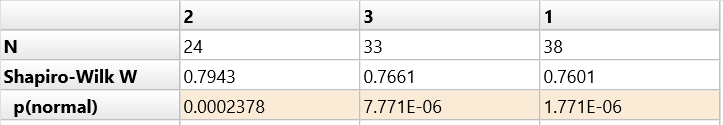
scaled_skull_length, scaled_skull_height, scaled_skull_width, scaled_naris_to_anterior_margin, scaled_naris_to_dorsal_margin, scaled_orbit_to_dosal_magin, K_means_cluster (group)

**Settings:**

NA

**Test:** Welch *F* test - (k-means)

**Inputs:**

scaled_skull_length, scaled_skull_height, scaled_skull_width, scaled_naris_to_anterior_margin, scaled_naris_to_dorsal_margin, scaled_orbit_to_dosal_magin, K_means_cluster (group)

**Settings:**

NA

**
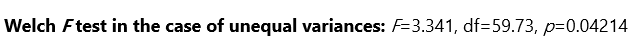
Results:**
